# Proangiogenic and Collagen-Promoting Effects of a 70% Ethanol Extract of *Grateloupia angusta* in Cutaneous Wound Models

**DOI:** 10.3390/ijms27073138

**Published:** 2026-03-30

**Authors:** Seongtae Jeong, Seahyoung Lee, Bomi Kim, Hanbyeol Moon, Hojin Kim, Myung Hun Yeon, Jung-Won Choi, Sang Woo Kim, Il-Kwon Kim, Byeong-Wook Song, Gyoonhee Han, Soyeon Lim

**Affiliations:** 1 The Interdisciplinary Graduate Program in Integrative Biotechnology, Yonsei University, Seoul 03722, Republic of Korea; 91seongtae@gmail.com; 2 Institute of Translational Aging Research, Catholic Kwandong University International St. Mary’s Hospital, Incheon Metropolitan City 22711, Republic of Korea; sam1017@ish.ac.kr (S.L.);; 3 Department of Convergence Science, College of Medicine, Catholic Kwandong University, Gangneung-si 25601, Republic of Korea; 4 Medical Science Research Institute, College of Medicine, Catholic Kwandong University, Incheon Metropolitan City 22711, Republic of Korea; 5 Department of Integrated Omics for Biomedical Sciences, Graduate School, Yonsei University, Seoul 03722, Republic of Korea; 6 Department for Medical Science, College of Medicine, Catholic Kwandong University, Gangneung-si 25601, Republic of Korea; 7 Marine. Inc., 38 Heungan-daero, 427 Beon-gil, Dongan-gu, Anyang-si 14059, Republic of Korea

**Keywords:** *Grateloupia angusta*, marine extract, wound healing, endothelial cells, dermal fibroblasts, Raw264.7

## Abstract

Marine red algae have been reported to contain a variety of bioactive compounds that are effective in promoting wound-healing processes. In the present study, the wound-healing potential of *Grateloupia angusta*, which has been rarely explored, was examined using in vitro and in vivo models. A 70% ethanol extract of *G. angusta* (GAE) was prepared and profiled by liquid chromatography–mass spectrometry (LC-MS). Its effects on the wound-healing process were examined using three different types of cells that participate in this process, namely, Raw264.7, human umbilical vein endothelial cells (HUVECs), and human dermal fibroblasts (HDFs). Various assays including migration/scratch, tube formation, procollagen type I C-peptide production, and Western blotting were used to investigate the therapeutic potential of GAE. In vivo efficacy was tested in a mouse full-thickness skin incision wound model. In HUVECs, GAE increased viability, migration, tube formation, and vascular endothelial growth factor (VEGF) expression. Raw264.7 cells also showed increased VEGF production following GAE treatment. In HDFs, GAE did not affect proliferation and migration, but did increase collagen production. In mice, GAE accelerated wound closure from day 3 to day 5 and increased granulation/matrix with higher proliferating cell nuclear antigen (PCNA) and cluster of differentiation 31 (CD31) expression after a single topical application. In addition, keratin 14 (K14) expression was restored in GAE-treated wound tissues, suggesting improved epidermal re-epithelialization. Taken together, GAE promotes matrix production and pro-angiogenic activity in vitro and improves early wound repair in vivo, suggesting that *G. angusta* is a promising marine-derived candidate for wound-healing adjuvants. The results of the present study support further bioassay-guided fractionation and mechanistic validation in future studies.

## 1. Introduction

Wound healing is a continuous and coordinated multistep physiological process (hemostasis, inflammation, proliferation, and remodeling) in which various cells, cytokines, and growth factors are involved [1]. In the hemostasis phase, exposed collagen from the cutaneous injury activates intrinsic and extrinsic coagulation pathways, provoking platelet aggregation and transient vasoconstriction that limits bleeding and induces a hypoxic milieu with a glycolytic shift and local pH changes [2]. The ensuing fibrin clot functions as a provisional matrix that recruits platelets and leukocytes; the platelets trapped within the clot degranulate and release mediators that provide antimicrobial defenses and orchestrate cytokine and growth factor signaling to initiate tissue repair [3]. The inflammatory phase—a largely innate immune response—overlaps temporally with hemostasis and represents the early stage of wound healing. Neutrophils rapidly infiltrate the wound, followed by monocytes that differentiate into macrophages to clear microbes and cellular debris. Classically activated Type I macrophages (M1) amplify the response by secreting key cytokines and growth factors, such as interleukin-1β (IL-1β) and tumor necrosis factor-α (TNF-α) [4]. As the inflammatory phase transitions toward resolution, alternatively activated Type II macrophages (M2) produce pro-repair mediators and growth factors, such as vascular endothelial growth factor (VEGF), that support the subsequent proliferative phase [5,6]. During proliferation, fibroblasts, keratinocytes, and endothelial cells drive granulation tissue formation, re-epithelialization and neo/revascularization. These processes are facilitated by heightened VEGF signaling [7]. Fibroblasts migrate into the wound bed, proliferate, and deposit extracellular matrix (ECM), with type I collagen constituting the predominant structural component under homeostatic condition [8]. Keratinocytes—the principal cells of the epidermis—re-epithelialize the wound by proliferating at the edges, differentiating, and migrating over the provisional matrix to restore barrier continuity. Endothelial cells, activated by paracrine cues from keratinocytes and fibroblasts (including VEGF), invade the granulation tissue and assemble nascent capillary networks that re-establish perfusion [9]. Finally, during remodeling, as type III collagen is replaced by type I collagen, the composition of the ECM progressively changes to increase tensile strength and facilitate restoration of tissue function [10].

A broad spectrum of interventions have been used or are under development for wound care, ranging from conventional measures (topical anti-bacterial agents for superficial wounds and moisture-retentive wound dressing) to advanced approaches such as negative pressure wound therapy, hyperbaric oxygen, multilayer dressing, nanomedicine, and photonics/low temperature plasma modalities. Despite such progress, important limitations persist. Biofilms compromise antibiotic efficacy, and growth factor therapeutics are quickly inactivated in the chronic wound milieu. Drug delivery from medicated dressings is often suboptimal at the target tissue [11,12]. Furthermore, nanocarriers pose a risk of cytotoxicity/accumulation, unpredictable release, and poor biofilm penetration, and cold plasma has a narrow therapeutic window, device variability, and high costs [13,14].

In parallel, natural product-based strategies, particularly marine algae-derived biomaterials and extracts, are being explored as adjuvants or actives that can accelerate repair while potentially reducing adverse effects associated with purely synthetic agents [15]. Historically, marine algae have been applied to wound care owing to their extraordinary antioxidant, anti-inflammatory, and antimicrobial properties [16]. Consistent with this, multiple studies have reported that algal extracts enhanced cell proliferation and migration, and polysaccharides such as alginate, carrageenan, and fucoidan exhibited therapeutic benefits and are already in clinical use for wound management [17,18]. Recent reviews and meta-analysis further underscore the growing preclinical and clinical support for algae-derived dressings and therapeutics, reinforcing their promise as complementary wound-healing interventions [18,19].

*Grateloupia angusta*, a halymenialean red alga, is widely distributed and is regarded as one of the common subtidal seaweed species in Korea and Japan [20]. As a member of the genus *Grateloupia*, it belongs to a group of red macroalgae that has attracted increasing interest as a source of bioactive marine metabolites and functional biomaterials. However, compared with other red algae, the chemical and pharmacological properties of *G. angusta* remain only sparsely investigated. The currently available evidence is limited but suggests potential relevant to tissue repair. For example, Yoon et al. reported that a *G. angusta* extract (GAE; 100 μg/mL) increased collagen and hyaluronic acid production by more than 140% and 120%, respectively [21]. In addition, an ethanol extract of *Grateloupia elata*, a member of the genus *Grateloupia*, showed significant antioxidant effects in human fibroblasts [22]. Collectively, these findings suggest that *Grateloupia* species may contain bioactive constituents capable of modulating extracellular matrix metabolism and wound-related cellular responses. At the same time, the limited species-specific literature on *G. angusta* highlights an important knowledge gap and underscores the need to investigate its potential in wound repair.

Based on this background, we hypothesized that a 70% ethanol extract of *G. angusta* could beneficially modulate multiple processes involved in cutaneous wound healing. To test this hypothesis, we evaluated its effects on three representative cell types that are involved in repair, namely, macrophage-like Raw264.7 cells, human umbilical vein endothelial cells (HUVECs), and human dermal fibroblasts (HDFs). In addition, its wound-healing activity was further assessed in vivo using a mouse model of cutaneous injury.

## 2. Results

### 2.1. Chemical Characterization of the GAE Extract

LC-MS analysis was conducted to identify the chemical constituents of the GAE extract. The resulting chromatogram is shown in Figure S1, and the identified chemical components are listed in Table S1.

### 2.2. GAE Stimulates VEGF Production in Raw264.7 Cells

**Figure 1:**
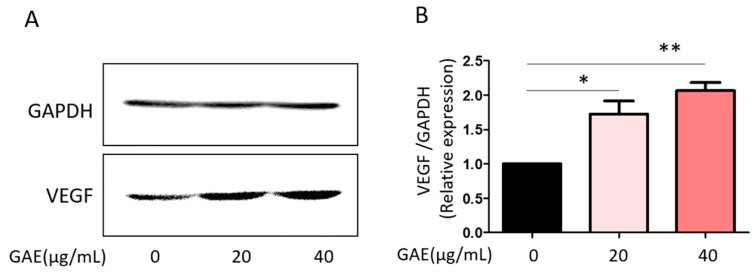
VEGF expression level of *Grateloupia angusta* extract (GAE)-treated Raw264.7 cells. (**A**) VEGF protein expression was analyzed by Western blot in Raw264.7 cells treated with GAE at concentrations of 20 μg/mL and 40 μg/mL. GAPDH was used as the loading control for normalization. (**B**) Densitometric quantification of VEGF expression is presented as a bar graph. Data are expressed as mean ± SEM (*n* ≥ 3). * *p* < 0.05, ** *p* < 0.01 versus control.

To determine whether GAE induces VEGF expression, Raw264.7 cells were treated with GAE at concentrations of 20 μg/mL and 40 μg/mL. VEGF is a key regulator of the proliferative phase of wound healing, promoting the proliferation of epithelial cells, vascular endothelial cells, and fibroblasts. Western blot analysis indicated that GAE increased VEGF protein levels in a concentration-dependent manner (Figure 1).

### 2.3. GAE Increases Proliferation, Migration, and Tube Formation of HUVECs

**Figure 2:**
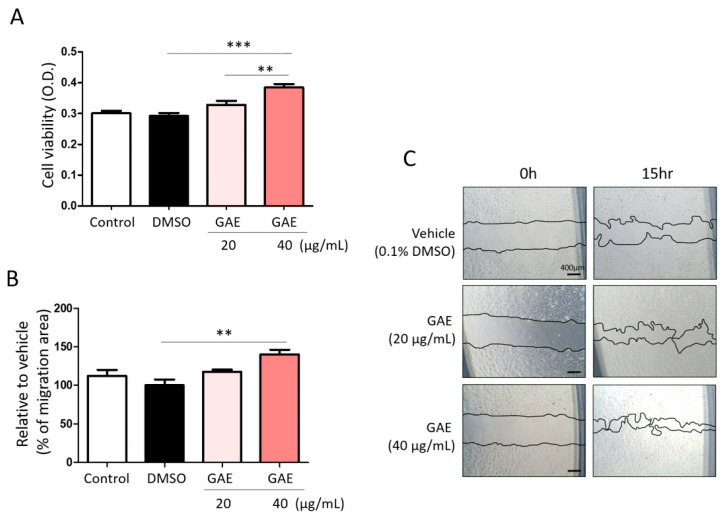
The effect of GAE on cell viability and migration in HUVECs. (**A**) Cell viability measured by WST-1 assay after 24 h of treatment with GAE (20 μg/mL and 40 μg/mL), shown as a bar graph. (**B**) Quantification of cell migration area (%) following treatment with 20 μg/mL and 40 μg/mL GAE for 15 h, presented as a bar graph. (**C**) Representative wound-healing images of HUVECs at 0 and 15 h post-treatment. Data are presented as mean ± SEM (*n* ≥ 3). ** *p* < 0.01, *** *p* < 0.001. DMSO: dimethyl sulfoxide.

**Figure 3:**
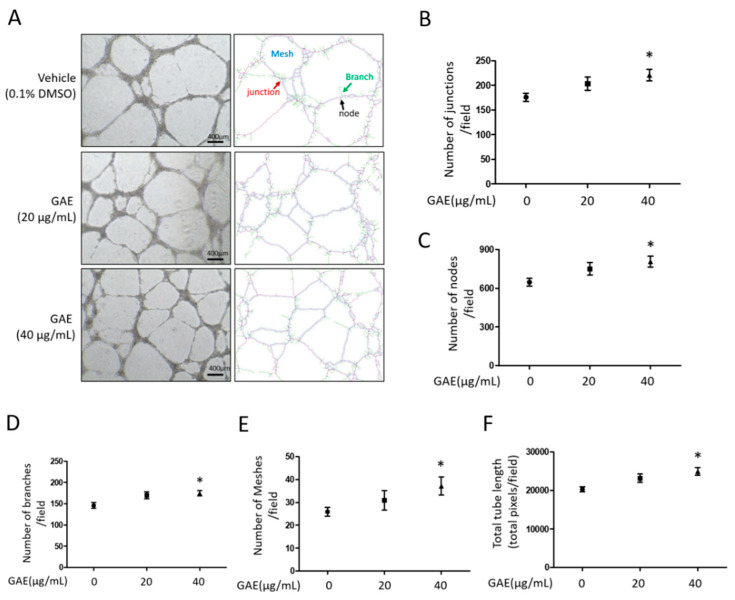
GAE promotes tube formation in HUVECs. (**A**) Representative images of tube formation in HUVECs treated with vehicle (0.1% DMSO) or GAE (20 μg/mL and 40 μg/mL) after 16 h of incubation on Matrigel. Tube structures were visualized (**B**–**F**) and quantitative analysis was performed using ImageJ 1.52a with the Angiogenesis Analyzer plugin. Junctions (red), nodes (black), branches (green), and meshes (blue) were identified using color-coded overlays. Tube formation parameters were quantified and are shown as bar graphs. Data are presented as mean ± SEM (*n* ≥ 3). * *p* < 0.05 vs. vehicle. DMSO: dimethyl sulfoxide.

**Figure 4:**
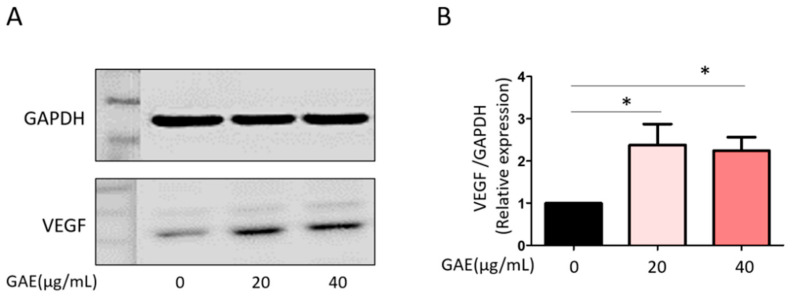
VEGF expression in GAE-treated HUVECs. (**A**) VEGF protein levels were analyzed by Western blot in HUVECs treated with GAE at concentrations of 20 μg/mL and 40 μg/mL. (**B**) Relative VEGF expression was quantified by densitometry and is presented as a bar graph. Data were normalized to GAPDH and expressed as mean ± SEM (*n* ≥ 3). * *p* < 0.05 vs. control.

To evaluate the effects of GAE on endothelial cell viability, HUVECs were treated with GAE at concentrations of 20 μg/mL and 40 μg/mL. GAE significantly increased the cell viability to 112.2 ± 10.58% and 117.2 ± 9.08%, respectively, compared to the untreated control (Figure 2A). The migratory capacity of HUVECs was subsequently assessed using a wound-healing assay. GAE treatment enhanced cell migration at the 40 μg/mL concentration, showing a significantly smaller uncovered wound area compared to control group (Figure 2B,C). To further determine the pro-angiogenic potential of GAE, in vitro tube formation assays were performed. Cells were seeded and treated with GAE (20 μg/mL and 40 μg/mL), and tube-like structures were analyzed at the indicated time points. Quantitative analysis revealed a concentration-dependent increase in key parameters, including the number of junctions, nodes, branches, and meshes, and total tube length (Figure 3). Additionally, Western blot analysis revealed that GAE treatment significantly upregulated VEGF protein expression at both 20 μg/mL and 40 μg/mL concentrations, suggesting that increased VEGF production is responsible for its pro-angiogenic effects (Figure 4).

Collectively, these results demonstrate that GAE promotes angiogenic activity in HUVECs by enhancing proliferation, migration, tube formation, and VEGF expression.

### 2.4. GAE Promotes Collagen Synthesis in HDFs

**Figure 5:**
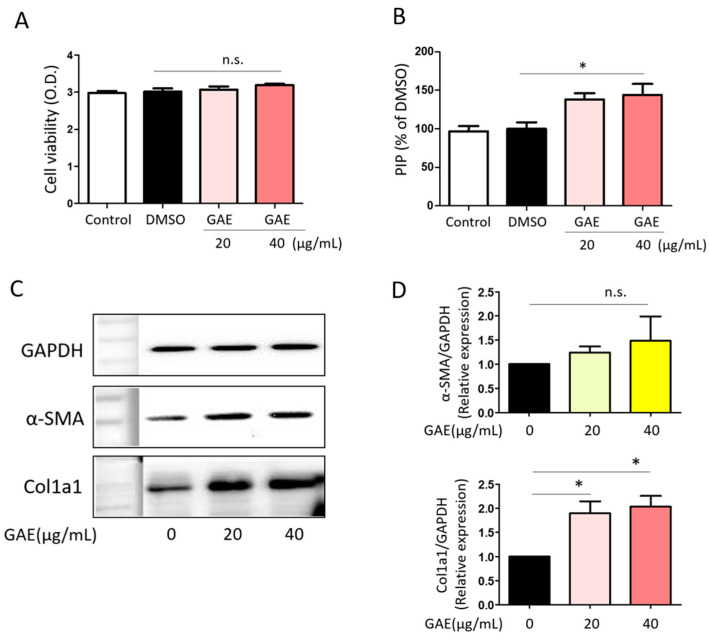
Effects of GAE on viability, extracellular matrix production, and fibrotic protein expression in HDFs. (**A**) Cell viability of HDFs was assessed after treatment with GAE (20 µg/mL and 40 µg/mL). (**B**) The concentration of procollagen type I C-peptide (PIP) in the culture supernatant was measured to evaluate collagen synthesis following GAE treatment. (**C**) Protein expression levels of α-SMA and Col1a1 were analyzed by Western blot following GAE treatment. (**D**) Densitometric quantification of α-SMA and Col1a1 expression, normalized to GAPDH, is presented as a bar graph. All data are shown as mean ± SEM (*n* ≥ 3). * *p* < 0.05; n.s., not significant. DMSO, dimethyl sulfoxide.

To evaluate the effect of GAE on human dermal fibroblast (HDF) viability, the cells were treated with GAE at concentrations of 20 µg/mL and 40 µg/mL for 24 h. The WST-1 assay results showed no significant change in cell viability at either concentration, indicating that GAE does not promote HDF proliferation (Figure 5A). Next, we assessed the effect of GAE on collagen synthesis by measuring the concentration of procollagen type I C-peptide (PIP) in the culture supernatant. GAE treatment increased PIP levels in a dose-dependent manner, with a statistically significant increase at 40 µg/mL (Figure 5B). To further explore the fibrotic response, the expression levels of α-SMA and Col1a1 were analyzed by Western blot. While α-SMA expression showed a non-significant increasing trend with GAE treatment, Col1a1 expression was significantly upregulated at both 20 µg/mL and 40 µg/mL (Figure 5D). Given the role of fibroblast migration in wound healing, we also performed a wound-healing assay to determine whether GAE affects the migratory capacity of HDFs. However, no significant changes in migration area were observed following GAE treatment, suggesting that GAE does not influence HDF migration.

### 2.5. GAE Accelerates Wound Healing and Enhances Angiogenesis, Proliferation, and Epidermal Regeneration Marker Expression In Vivo

**Figure 6:**
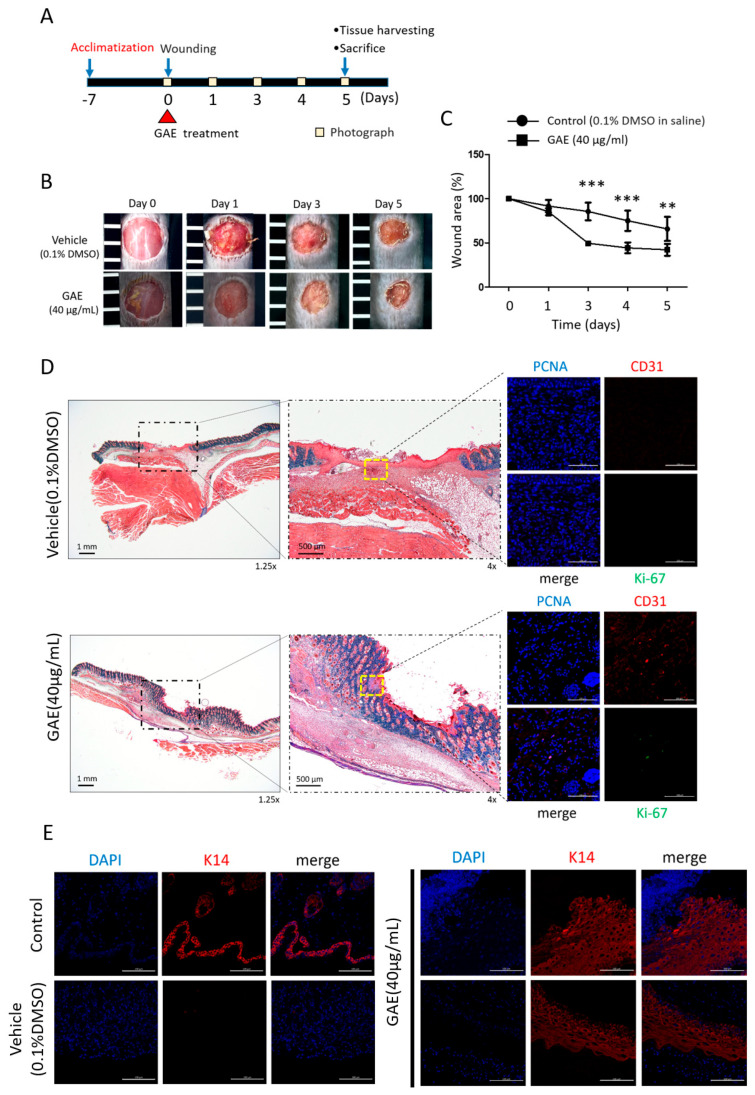
GAE promotes wound healing in mice. (**A**) Schematic of the in vivo wound-healing experiment. (**B**) Representative images of wounds captured daily after injury and treatment with either vehicle or GAE. The spacing between the adjacent scale bars is 5 mm. (**C**) Quantification of wound area over time, presented as a bar graph comparing vehicle- and GAE-treated groups. Data are expressed as mean ± SEM (*n* = 3). *** *p* < 0.001, ** *p* < 0.01 vs. vehicle. (**D**,**E**) Masson’s trichrome staining of full-thickness skin wounds at day 5 post-injury. Left: low-magnification (1.25×) images showing overall wound structure. Scale bar: 1 mm. Right: higher magnification (4×) images of the boxed region. Scale bar: 500 µm. Immunofluorescence staining of adjacent tissue sections was performed to detect PECAM-1/CD31(APC, red), Ki-67 (FITC, green), K14 (rhodamine, red) and nuclei (DAPI, blue), imaged at 20× magnification. Scale bar: 100 µm. DMSO, dimethyl sulfoxide.

To assess the effect of GAE on cutaneous wound healing, a single topical application was administered immediately after wound induction, and wound closure was monitored over a 5-day period (Figure 6A). On day 1, there was no statistically significant difference in wound area between the vehicle- and GAE-treated groups. However, from day 3 onwards, GAE-treated mice exhibited significantly faster wound closure compared to the controls, and this difference was maintained until day 5 (Figure 6B,C).

To further investigate the underlying mechanisms, wound tissues collected on day 5 were subjected to immunohistochemical and immunofluorescence analyses. Masson’s trichrome staining showed enhanced granulation tissue formation in the GAE-treated group. Additionally, immunostaining revealed increased expression of PCNA and CD31 in the dermal layer of GAE-treated wounds, indicating increased proliferative activity and neovascularization, respectively (Figure 6D). To assess epidermal restoration, keratin 14 (K14) expression was examined in skin sections. K14 expression was scarcely detected in wound skin tissues, whereas it was clearly observed in normal skin and markedly restored in GAE-treated wound tissues (Figure 6E). These findings suggest that GAE facilitates tissue regeneration by promoting dermal proliferative responses, vascular remodeling, and epidermal re-epithelialization during the proliferative phase of wound healing.

## 3. Discussion

This study demonstrates that the 70% ethanol extract of *G. angusta* (GAE) engages multiple cellular axes relevant to the proliferative phase of wound repair. In endothelial cells, GAE consistently enhanced angiogenic activities including migration, network formation, morphological changes, and VEGF expression. Also, macrophage-like Raw264.7 cells exhibited increased VEGF production following GAE treatment, providing a paracrine environment favorable to neovascularization. In fibroblasts, GAE induced the expression of the extracellular matrix protein Col1A1 without inducing proliferation or altering migration, indicating that its effect is matrix-centric rather than mitogenic. Furthermore, although proliferation of HDFs is important in wound-healing process, excessive proliferation of HDFs can lead to keloid formation [23]. Thus, these findings suggest that GAE preferentially enhances extracellular matrix production without driving a hyperproliferative or strongly myofibroblast-dominant phenotype, which may be advantageous for improving tissue quality while minimizing the risk of pathological scarring. On the other hand, GAE significantly increased the proliferation and migration of endothelial cells with enhanced VEGF production. This strongly suggests that GAE can promote regeneration-accelerating mechanisms such as angiogenesis in non-tumor wound environments and has therapeutic potential, particularly in chronic wounds characterized by an insufficient blood supply, such as diabetic foot ulcers and ischemic or venous leg ulcers [24,25]. This interpretation is broadly consistent with previous reports showing that red algal extracts and marine-derived wound-healing materials can modulate collagen production, angiogenesis, and inflammatory signaling in wound-related cellular systems [18,19,21].

In this study, the in vitro findings were further supported by the in vivo outcomes. The results of our animal study demonstrated that a single topical application of GAE significantly accelerated wound closure from day 3 onwards until day 5. Histological and immunostaining analyses further supported this in vivo effect. Masson’s trichrome staining showed enhanced granulation tissue formation in GAE-treated wounds, while PCNA and CD31 expression was increased in the dermal layer, indicating enhanced proliferative activity and neovascularization, respectively. In addition, keratin 14 (K14) staining was scarcely detected in wounded skin tissues, whereas it was clearly observed in normal skin and markedly restored in GAE-treated wounds. Because K14 is a marker associated with basal keratinocytes and epidermal integrity [26], these findings suggest that GAE may also promote epidermal restoration/re-epithelialization during cutaneous wound repair. These in vivo findings are also in agreement with previous preclinical studies indicating that marine-derived materials can promote granulation tissue formation, angiogenesis, and tissue regeneration during cutaneous wound healing [17,18,27,28]. However, the efficacy of the regimen used needs to be highlighted. For this particular study, a single topical application of GAE was used, and it was able to reduce the wound size by up to approximately 50% within the first 3 days. Nevertheless, the reduction in the wound size gradually decreased to approximately 20 and 10% on days 4 and 5, respectively, suggesting that the efficacy of GAE rapidly decreases after day 3. Such a gradual decrease in efficacy seems to be common so many previous preclinical studies used repeated-treatment regimens with 2–4-day intervals for wound care [29,30,31]. Taken together, these data imply that an every-three-day (q3d) dosing may be necessary to maximize the benefits of the regimen. Accordingly, our subsequent studies will adopt a q3d treatment schedule to test whether repeated treatment can preserve and fortify the early wound-closure effect of GAE.

For the present study, GAE was prepared using an ethanol:water (70:30 *v/v*) system, which was demonstrated to be effective in facilitating high recovery of flavonoids and total polyphenols from the red algae *Gelidium sesquipedale*, a congeneric species of *Grateloupia* [32]. Polyphenols from marine macroalgae are widely associated with various pharmacological activities such as antioxidant, anti-inflammatory, and anti-bacterial effects [33]. We did not quantitatively evaluate the amount of polyphenols in the present work. However, another study used the identical GAE obtained from the MABIK and indicated that it had a total polyphenol content of 19.10 ± 0.15 mg phloroglucinol equivalent (PGE/g), although the GAE extraction did not show strong radical-scavenging effects against DPPH, superoxide, and hydroxyl radicals [34]. Regarding the role of reactive oxygen species (ROS) in the early phase of wound repair, it has been reported that physiological levels of ROS are required to support host defense and drive regeneration [35]. On the other hand, it also has been reported that excessive ROS levels can lead to oxidative stress and chronic wound formation [36]. These studies indicate that ROS in the wound repair acts as a double-edged sword so that an appropriate level of ROS is necessary, but excessive ROS levels exacerbate the wound. Therefore, given that a certain level of reactive oxygen species (ROS) is required for the early inflammatory and signaling events in wound healing, the relatively weak radical-scavenging capacity of GAE might be advantageous as it could avoid excessive suppression of ROS while still supporting matrix deposition and vascularization in the later phases of repair.

Regarding the collagen-forming capacity of red algae, a previous study showed that an ethanol extract of *Grateloupia elata* showed higher polyphenol and flavonoid contents (29.208 ± 0.396 mg gallic acid equivalent (GAE/g) and 29.134 ± 1.234 mg quercetin equivalent (QE/g)), and a significant antioxidant activity emerged at concentrations of 1000 µg/mL to 5000 µg/mL and collagen synthesis (PIP amount) was increased 1.69-fold at 100 µg/mL and 2.45-fold at 1000 µg/mL compared to the control in CCD-986sk human skin fibroblasts [22]. In our study, 40 µg/mL of GAE increased PIP and collagen I levels by 1.43- and 2.03-fold compared with the vehicle control, respectively (Figure 5B,D). These results suggest that the collagen-forming capacity of *G. angusta* is higher than that of *G. elata* in our human dermal fibroblast model.

The roles of the bioactive phytochemicals of *G. angusta* have only been sparsely characterized. Our LC-MS analysis identified few putative bioactive compounds such as galactosylglycerol and monoglyceride species (MG (16:0; monopalmitin)) (Table S1). Prior work showed that galactosylglycerol promotes re-epithelialization in HaCaT keratinocytes via ERK1/2 MAP kinase and intracellular calcium signaling [37]. Consistently, the related red-algal glycerol galactoside floridoside suppresses NF-κB/MAPK-driven inflammatory responses [38], and glycerol itself exerts anti-irritant and anti-inflammatory effects in keratinocyte models [39]. Although mechanistic data for monoglycerides in skin repair are limited, they have been demonstrated to be anti-bacterial agents that destabilize bacterial cell membranes, suggesting that monoglycerides, as antimicrobial lipids, may indirectly support wound repair by inhibiting biofilm formation [40]. Another compound identified from the LC-MS analysis in our study is choline. Choline has been reported to have therapeutic potential in wound healing. Banerjee et al. demonstrated that a choline-containing hydrogel enhanced angiogenesis in an in vitro model using HUVECs [41], chick embryos, and a rat ischemic stroke model [27,42]. Nevertheless, to better understand the underlying mechanisms of these putative bioactive compounds, further studies are required.

As conventional wound therapies face the persistent side-effect issues described earlier, interest is growing in marine algae-derived materials and extracts as adjuvants or alternatives. Marine macroalgae have provided some clinically usable biomaterials such as sulfated polysaccharides and phenolic-rich extracts with antioxidant, anti-inflammatory, antimicrobial, and pro-angiogenic properties, and they facilitate multiple repair process with potentially fewer adverse effects than purely synthetic agents [28,43]. Nevertheless, for algae-based interventions including GAE, further work is necessary to prove or disprove their therapeutic potential.

The present study has several limitations. First, published studies specifically investigating *Grateloupia angusta* extract remain limited, particularly in the context of wound healing, angiogenesis, and collagen regulation, which prevented a direct comparison of our findings with previous species-specific reports. Accordingly, some parts of our interpretation were supported by studies on related red algal species or comparable marine-derived wound-healing materials. Second, the present in vivo study was performed with a small number of animals per group (n = 3) over a relatively short observation period in an acute wound model using healthy mice, which limits the statistical robustness and translational relevance of the findings. Therefore, the present in vivo results should be interpreted as preliminary proof-of-concept evidence. Third, the study lacks a positive control, which limited direct comparison of the efficacy of GAE against established wound-healing agents. In addition, only two concentrations of GAE were tested, which restricts a comprehensive evaluation of dose-dependent effects. Fourth, the study also did not include antimicrobial or biofilm-related outcomes. Fifth, although LC-MS profiling was performed, the identified metabolites were only tentatively annotated, and individual compounds were not isolated or functionally validated. Finally, apoptosis, oxidative stress, and cell cycle regulation were not directly examined. Therefore, although our data support the pro-regenerative activity of GAE at the functional level, the underlying cellular mechanisms remain to be more fully defined.

Further work should (1) apply bioassay-guided fractionation and develop quantitative chemical markers; (2) define the underlying mechanisms by examining apoptosis, oxidative stress, and cell cycle regulation, together with validation using pathway inhibitors or neutralizing antibodies; (3) extend the scope of in vivo testing (large cohorts, dose–response analysis, repeated dosing, safety, clinically relevant impaired wound-healing models, such as diabetic, ischemia, infected, or compromised wounds); (4) include appropriate positive controls, such as established wound-healing agents or standardized active fractions, to allow for a direct comparison of efficacy; and (5) evaluate antimicrobial and biofilm effects and optimize delivery. These efforts will help identify the active compounds, refine the underlying mechanisms, and improve the translational potential of GAE.

## 4. Materials and Methods

### 4.1. Preparation of Grateloupia Angusta Extract (GAE) and Its Compounds

The 70% ethanol extract of *Grateloupia angusta* (MABIK NP3020004) was obtained from the National Marine Biodiversity Institute of Korea (MABIK, Seocheon-gun, Chungcheongnam-do, Korea). Specimens were collected in 2017 from Seogwipo, Jeju-do, Korea, under MABIK oversight. Freeze-dried whole seaweed of G. angusta (30 g) was first extracted by sonication in 70% ethanol (400 mL) using a water bath maintained at 22–25 °C (40 kHz for 1 h), and then heated under reflux for 1 h; this sequence was repeated three times and the extracts were pooled. The combined solution was filtered and concentrated in vacuo (rotary evaporation). The extraction yield [(dry extract (g))/(dry sample (g)) × 100] was 2.67% (*w*/*w*). The dried extract (800 mg dry weight) was preserved at −80 °C until use and freshly dissolved in dimethyl sulfoxide (DMSO) for in vitro assays.

### 4.2. Cell Culture

Raw264.7 macrophages. Raw264.7 cells (TIB-71; ATCC, Manassas, VA, USA) were maintained in Dulbecco’s modified Eagle’s medium (DMEM) (30-2002; ATCC) containing 10% fetal bovine serum (FBS) (16000-044, Thermo Fisher Scientific, Waltham, MA, USA), 100 U/mL penicillin, and 100 μg/mL streptomycin (15140-122; Thermo Fisher Scientific).

Human umbilical vein endothelial cells (HUVECs). HUVECs (C2519A, Lonza, Allendale, NJ, USA) were cultured in endothelial basal medium-2 (EBM-2; CC-3156, Lonza) supplemented with the EGM-2 SingleQuot BulletKit (CC-4176, Lonza) and plated on dishes pre-coated with 1.5% gelatin.

Human dermal fibroblasts (HDFs). HDFs (CB-HF-003; Cefo Co., Ltd., Seoul, Korea) were grown in DMEM (11965-092, Thermo Fisher Scientific) supplemented with 10% heat-inactivated FBS and 1% penicillin–streptomycin.

All cell types were incubated at 37 °C in a humidified 5% CO_2_ atmosphere; media were changed every 2 days, and cells at passage 10 or below were used for experiments.

### 4.3. Evaluation of Cell Viability (WST-1)

The cytotoxicity of GAE was assessed using a water-soluble tetrazolium 1 (WST-1) assay. Cells were exposed to GAE and viability was quantified with the EZ-Cytox WST assay kit (EZ-3000; Dogenbio, Seoul, Korea) according to the manufacturer’s instructions. Absorbance at 450 nm was recorded using a Multiskan FC microplate reader (Thermo Fisher Scientific).

### 4.4. Wound-Healing Assay (Migration)

A scratch (wound-healing) assay was performed in HUVECs and HDFs. Cells were seeded at a density of 5 × 10^4^ cells per well in 24-well plates and cultured in complete medium until confluent. Monolayers were serum-starved in basal medium for 24 h, after which, a linear scratch was made with a sterile 200 μL pipette tip. Cells were then treated with GAE (20 and 40 μg/mL) in basal medium containing 0.5% FBS. Phase-contrast images were captured at 0 h and 15 h using an inverted microscope (CKX41; Olympus, Tokyo, Japan) equipped with a digital camera (eXcope T300; Olympus). Wound closure was quantified from image sets using ImageJ 1.52a (NIH, Bethesda, MD, USA).

### 4.5. Tube Formation in HUVECs

A tube formation assay was conducted according to the manufacturer’s instructions (Corning, NY, USA). Briefly, Matrigel (10 mg/mL; Corning) was dispensed into 24-well plates and allowed to polymerize. HUVECs (1 × 10^5^ cells/well) were then seeded and treated with GAE (20 and 40 μg/mL). After 16 h, phase-contrast images were acquired using an inverted microscope (CKX41, Olympus) equipped with a digital camera (eXcope T300; Olympus). Quantitative analysis of tube networks was performed in ImageJ (Angiogenesis Analyzer) software, yielding standard angiogenic metrics (number of junctions, nodes, branches, and meshes, and total tube length) [44].

### 4.6. Quantification of Procollagen Type I C-Peptide (PIP)

Procollagen type I C-peptide (PIP) was quantified in culture supernatants to assess collagen synthesis. HDFs were plated at a density of 5 × 10^4^ cells per well in 96-well plates and allowed to attach, then serum-deprived for 24 h. The cells were treated with GAE (20 μg/mL and 40 μg/mL) in serum-free medium for 48 h. Supernatants were collected, clarified by centrifugation (3000 rpm, 3 min, 4 °C), and analyzed using a PIP EIA Kit (MK101; Takara Bio, Shiga, Japan) according to the manufacturer’s instructions. Absorbance was read at 450 nm using a microplate reader (Multiskan FC; Thermo Fisher Scientific).

### 4.7. Western Blot

Proteins were extracted in RIPA buffer supplemented with protease inhibitor (sc-11697498001; Santa Cruz, Dallas, TX, USA) and phosphatase inhibitors (4906845001; Thermo Fisher Scientific). Lysates were quantified by the Bicinchoninic Acid (BCA) assay, resolved on 12% SDS–polyacrylamide gels, and transferred to Immobilon-P PVDF membranes (IPVH00010; Merck Millipore, Burlington, MA, USA). The membranes were blocked in 5% skim milk (room temperature, 30 min) and incubated overnight at 4 °C with primary antibodies, followed by 1 h at room temperature with HRP-conjugated secondary antibodies in TBST (Tris-buffered saline, 0.1% Tween-20) containing 5% skim milk. Immunoreactive bands were visualized by enhanced chemiluminescence (ECL, AbClon, Seoul, Korea) and quantified using ImageJ 1.52a.

The antibodies and dilutions used were as follows: Primary antibodies: Col1A1 (1:500; ab34710, Abcam, Cambridge, UK), α-SMA (1:1000; ab7817, Abcam), VEGF (1:1000; ab46154, Abcam), and GAPDH (1:5000; sc-47778, Santa Cruz); Secondary antibodies: anti-mouse (1:2000; ADI-SAB-100-J, ENZO, Farmingdale, NY, USA) and anti-rabbit (1:2000; ADI-SAB-300-J, ENZO).

### 4.8. Animals and Cutaneous Wound Model

All animal experiments complied with ARRIVE 2.0 guidelines and were approved by the Institutional Animal Care and Use Committee of Catholic Kwandong University College of Medicine (CKU 01-2024-011). Male C57BL/6 mice (8 weeks, 20–25 g; Koatech, Pyeongtaek, Korea) were acclimated under standard laboratory conditions (12-h light/dark cycle, 25 ± 4 °C). The animals were randomized into two groups (n = 3 each): vehicle (0.1% DMSO) or GAE (40 μg/mL). Under isoflurane anesthesia, the dorsal hair was removed and the skin disinfected, then two full-thickness excisional wounds (15 mm diameter) were created on the mid-dorsal region using sterile scissors. Immediately after wound induction, a single topical dose of GAE or a vehicle was applied to the wound bed. Wounds were photographed on days 0, 1, 3, and 5, and areas were quantified in ImageJ. The percent wound area was calculated relative to the initial (day 0) wound area.

### 4.9. Histological and Immunohistochemical Evaluation of Cutaneous Wound Repair in Mice

Mice were euthanized by CO_2_ inhalation in accordance with institutional guidelines to minimize distress and discomfort. Death was confirmed and dorsal skin encompassing both full-thickness excisional wounds was harvested and fixed in 4% neutral-buffered formalin for histology.

Collagen deposition and extracellular matrix (ECM) remodeling were assessed using paraffin sections and Masson’s Trichrome (ScyTek, Logan, UT, USA), following the manufacturer’s instructions. The slides were examined with an Olympus BX53 light microscope and imaged using a DP27 digital camera (Olympus, Tokyo, Japan).

For immunohistochemistry, sections were deparaffinized, blocked with 2.5% normal horse serum for 1 h at room temperature. The sections were incubated overnight at 4 °C with primary antibodies against Ki-67 (AB9260, Millipore), CD31/PECAM-1 (sc-1506, Santa Cruz), and cytokeratin 14 (ab181595, Abcam) at a dilution of 1:100. After washing, APC-conjugated (sc-3860, Santa Cruz), FITC-conjugated (711-095-152, Jackson ImmunoResearch, West Grove, PA, USA), and rhodamine-conjugated (AP132R, Millipore) secondary antibodies were applied at a dilution of 1:100 for 1 h at room temperature; nuclei were counterstained with 4′,6-diamidino-2-phenylindole (DAPI; D21490, Thermo Fisher Scientific) at 1:2000 in the dark for 5 min. Images were acquired using an LSM 700 laser scanning confocal microscope (Carl Zeiss, Oberkochen, Germany).

### 4.10. Statistical Analysis

Statistical analyses were performed in GraphPad Prism 5 (GraphPad Software, San Diego, CA, USA). Experiments were repeated at least three times, and data are reported as the mean ± standard error of the mean (SEM). Comparisons between two groups used two-tailed Student’s t-test. For comparing more than three groups, one-way analysis of variance (ANOVA) with a Bonferroni post hoc test was used. A *p*-value < 0.05 was considered statistically significant.

## 5. Conclusions

Taken together, our results suggest that GAE facilitates wound healing by promoting fibroblast-driven extracellular matrix remodeling and angiogenic responses. These findings support the therapeutic potential of GAE as a bioactive marine-derived agent for enhancing cutaneous tissue repair. Nevertheless, further studies are warranted to identify the active compounds through bioassay-guided fractionation, verify the underlying mechanisms, and expand the dose–response evaluation as well as in vivo validation in larger cohorts and more clinically relevant impaired wound models, including chronic or compromised wound conditions. In addition, assessment of antimicrobial and antibiofilm activities, together with delivery optimization, will further strengthen the translational potential of GAE.

## Data Availability

The original contributions presented in this study are included in the article. Further inquiries can be directed to the corresponding author.

## Supplementary Materials

The following supporting information can be downloaded at: https://www.mdpi.com/article/10.3390/ijms27073138/s1.

## Abbreviations

The following abbreviations are used in this manuscript:

GAE	Grateloupia angusta extract
HUVEC	human umbilical vein endothelial cell
HDF	human dermal fibroblast
IL-1β	interleukin-1β
TNF-α	tumor necrosis factor-α
VEGF	vascular endothelial growth factor
ECM	extracellular matrix
PIP	procollagen type I C-peptide
α-SMA	alpha smooth muscle actin
Col1a1	collagen type I, alpha 1
PCNA	proliferating cell nuclear antigen
CD31	cluster of differentiation 31
K14	cytokeratin 14
ROS	reactive oxygen species
