# Supplementary material for: Proangiogenic and Collagen-Promoting Effects of a 70% Ethanol Extract of Grateloupia angusta in Cutaneous Wound Models
Source: Int J Mol Sci. 2026 Mar 30;27(7):3138. doi: 10.3390/ijms27073138 (PMC13073458; doi:10.3390/ijms27073138)

Supplementary Materials and Methods

Liquid Chromatography–Mass Spectrometry (LC-MS) Analysis

The phytochemical profile of GAE was analyzed by LC-MS to obtain an overview of putative constituents. The data were used for tentative annotation of major compound classes rather than for quantitative determination of individual metabolites. An aliquot of the Grateloupia angusta extract was prepared at 100 ppm in methanol (HPLC grade; Sigma-Aldrich) and analyzed on a Q Exactive Orbitrap plus mass spectrometer (Thermo Fisher Scientific) coupled to a Dionex Ultimate 3000 RSLC nano system. Chromatographic separation was achieved on an ACQUITY UPLC BEH C18 column (1.7 μm, 2.1 × 100 mm) maintained at 45 ℃, with a flow rate of 400 μL/min. Mobile phases were (A) 0.1 % formic acid in HPLC-grade water and (B) 0.1 % formic acid in HPLC grade acetonitrile. The Orbitrap was operated in positive ion mode with MS resolution 70,000 over an m/z range of 80-1000. Data-dependent MS/MS was acquired at 17,500 resolution, isolation window 2.0 m/z, and loop count of 5. Candidate elemental compositions and annotations were evaluated against the ChemSpider database (http://www.chemspider.com) and mzCloud MS library (http://www.mzcloud.org). Common plasticizers such as phthalate and sorbitol were treated as contaminants and excluded from further analysis.

Table S1. LC/MS profile of a 70 % ethanol *grateloupia angusta* extract (GAE)

No.	RT[min]	Formula	Candidate M.W.	Area (Max.)	Name by searching ChemSpider Results
1	17.184	$C_5 H_{10} O$	86.073	5734680973.2	Isovaleraldehyde
2	0.863	$C_9 H_{18} O_8$	254.099	3061550595.2	Galactosylglycerol
3	13.853	$C_{19} H_{38} O_4$	330.276	1737868951.3	MG(16:0/0:0/0:0)
4	0.824	$C_5 H_{13} N O$	103.099	1356394089.4	Choline
5	1.15	$C_5 H_7 N O_3$	129.042	712448075.2	L-Pyroglutamic acid
6	11.839	$C_{24} H_{30} O_6$	414.203	691335138.2	Bis(4-ethylbenzylidene)sorbitol
7	1.107	$C_4 H_7 N O$	85.052	659850822.8	2-Pyrrolidinone
8	14.393	$C_{24} H_{38} O_4$	418.308	568634155.8	Bis(3,5,5-trimethylhexyl) phthalate
9	0.966	$C_4 H_8 O_3 S$	136.019	554206912.8	Tetrahydrothiophene-3-ol 1,1-dioxide
10	1.609	$C_9 H_{19} N O_3$	189.136	527602732.6	1-[4-(2-Methoxyethoxy)tetrahydro-2H-pyran-4-yl]methanamine

Figure S1. LC-MS-based analysis and chemical structures of compounds 2-4 in the 70 % ethanol extract of Grateloupia angusta (GAE). A) LC-MS chromatograms of the blank (upper panel) and GAE sample (lower panel). B-D) MS spectra and UV profiles of compound 2 (B), 3 (C), and 4 (D) identified in the LC-MS analysis. Compound 1 was not detected under the present analytical conditions.

A

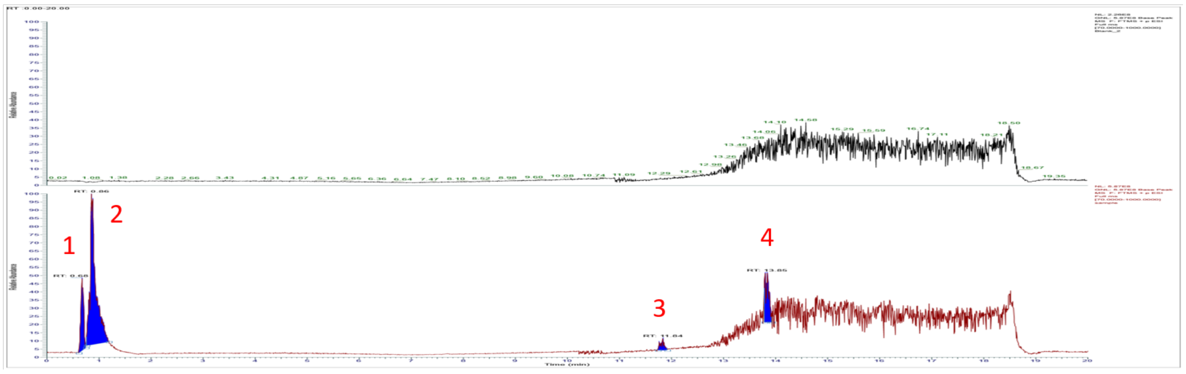

B

Compounds
08-1-2025 9:43

File name: 250106_JST

Study: New Study

Structure	Name	RT [min]	Formula	Calc. MW	Group Areas
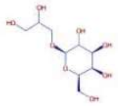	Galactosylglycerol	0.86	C9 H18 O8	254.09990	3.06e9

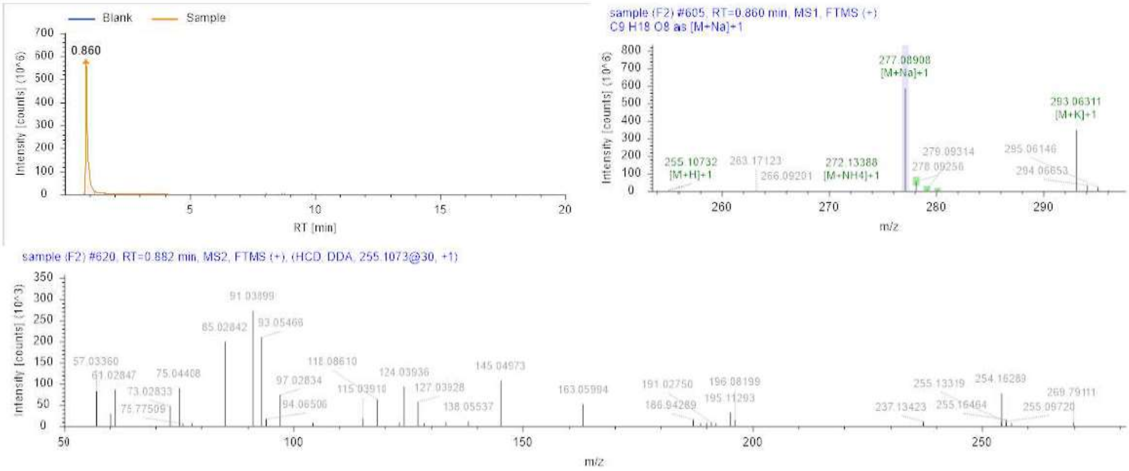

C

Compounds

08-1-2025 9:43

File name: 250106_JST

Study: New Study

Structure	Name	RT [min]	Formula	Calc. MW	Group Areas
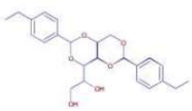	Bis(4-ethylbenzylidene)sorbitol	11.50	C24 H30 O6	414.20408	6.16e7

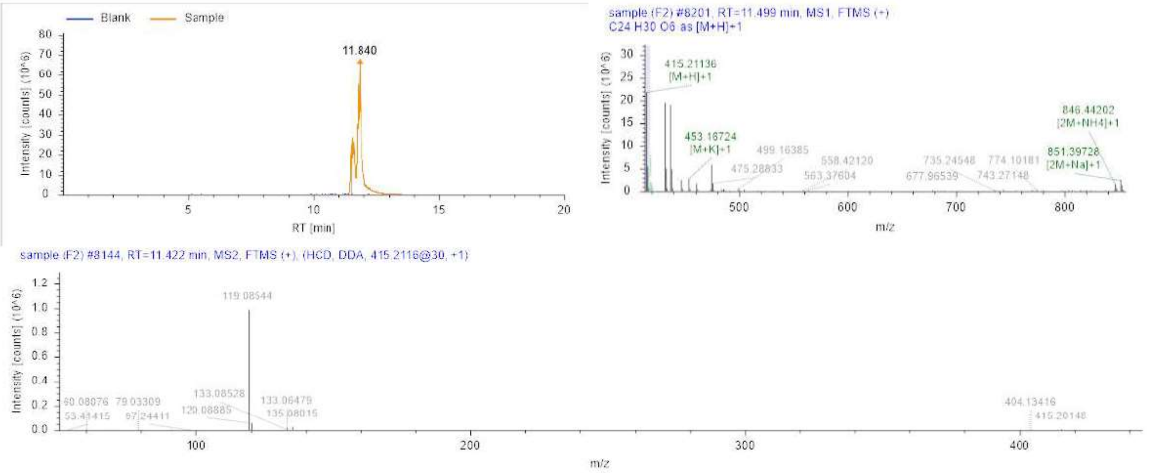

D

Compounds

08-1-2025 9:43

File name: 250106_JST

Study: New Study

Structure	Name	RT [min]	Formula	Calc. MW	Group Areas
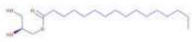	MG(16:0/0:0/0:0)	13.51	C19 H38 O4	330.27712	3.70e7

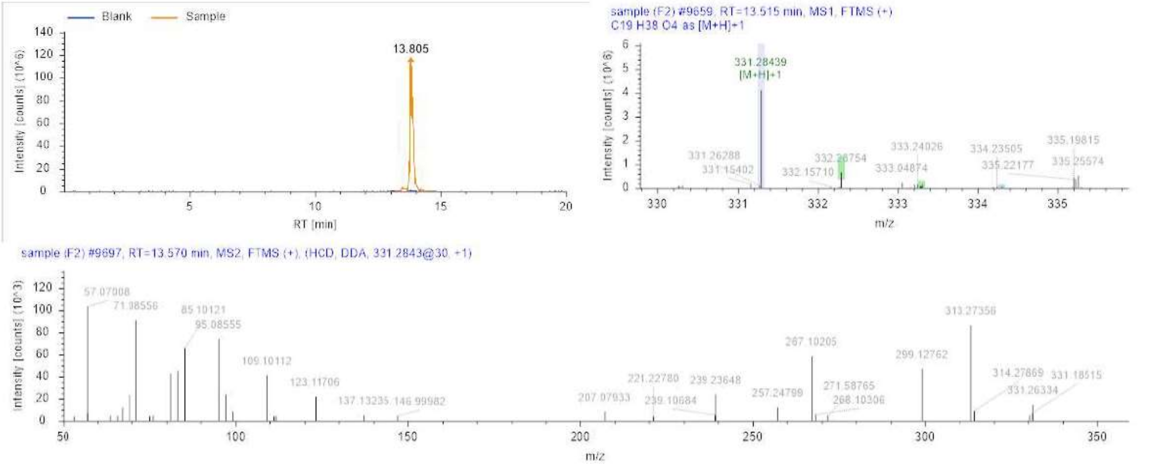

Supplement: Supplementary file 1 [file ijms-27-03138-s001.zip › ijms-4081868-supplementary.pdf]
